# Using Interpretative Phenomenological Analysis to Explore the Experiences of Mothers Who Have a Child With the Rare Genetic Disorder, *HNRNPU* ‐Related Neurodevelopmental Disorder: “Some Days, I Wear a Cape and I'm Super Mom and Some Days, I Have to Hide Under My Cape and I Can't Get Out for a Few Days”

**DOI:** 10.1002/jgc4.70310

**Published:** 2026-10-23

**Authors:** I. Overton, F. Docherty, M. Balasubramanian

**Affiliations:** ^1^ Division of Clinical Medicine, School of Medicine and Population Health University of Sheffield Sheffield UK; ^2^ Department of Clinical Genetics Sheffield Children's NHS Foundation Trust Sheffield UK

**Keywords:** advocacy, care responsibilities, HNRNPU, HNRNPU‐NDD, IPA, parent carer, parents experiences, psychological impact, rare disorder, ultra‐rare

## Abstract

HNRNPU‐related neurodevelopmental disorder is an ultra‐rare genetic condition characterised by developmental delay, intellectual disability, seizures and a range of complex health needs. Yet, little is known about how families experience and navigate life with this diagnosis. This study explored the lived experiences of seven mothers caring for a child with HNRNPU‐NDD, using Interpretive Phenomenological Analysis of semi‐structured interviews to gain an in‐depth understanding of how they manage with daily life and sustain their well‐being. Three group experiential themes (GETs) were generated: (1) From nurturer to care manager: how hypervigilance shapes daily life and maternal identity, (2) How mothers make sense of futures that no longer look as expected: grief, fear of future and reconstruction of hope, (3) Social worlds that ‘no longer fit’: experiences of isolation and disconnection. Together, these themes show the emotional duality, identity shifts and social challenges involved in raising a child with HNRNPU‐NDD. They are discussed alongside existing literature on rare disease caregiving, highlighting shared challenges as well as aspects that appear distinctive to ultra‐rare neurodevelopmental conditions. The findings illustrate that mothers require meaningful support acknowledging the emotional weight and relational shifts of caregiving, beyond the child's symptoms. This study aims to shed light into an under‐researched condition and offer suggestions for future research which should include broader family perspectives (fathers and siblings) and the development of support systems tailored to the complex needs of this population.

## Abstract (highlights)

**What Is Known About This Topic:**

Previous research has explored the emotional and psychological impact of caregiving in the context of chronic illness and genetic conditions, highlighting themes such as anticipatory grief, caregiver burden, and identity disruption. Studies using Interpretative Phenomenological Analysis (IPA) have shown how parents, particularly mothers, often experience shifts in self‐concept and social isolation as they navigate complex care responsibilities.

**What This Paper Adds to This Topic:**

This study offers the first qualitative exploration of parenting children with HNRNPU‐related neurodevelopmental disorder. This paper adds insight by identifying three experiential themes that deepen our understanding of maternal caregiving: the shift from nurturer to care manager driven by symptom hypervigilance; negotiating uncertain futures through grief, fear and hope; and the experience of social disconnection as families' realities change. These findings highlight the sustained psychological complexity of caregiving, the need for psychological safety in clinical interactions, and the importance of family‐centred support in genetic counseling practice.

## 1 Introduction

Rare genetic disorders are defined as those affecting approximately 1 in every 2000 people and ultra‐rare conditions impact less than 1 in 50,000 (European Union 2000; Smith et al. 2022). One such condition is *HNRNPU*‐related neurodevelopmental disorder (hereafter HNRNPU‐NDD), a neurodevelopmental disorder, with around only 300 cases reported worldwide (Balasubramanian 2022). This ultra‐rare disorder is caused by disruptions in the *HNRNPU* gene, as a result of chromosome 1q44 microdeletions and *de novo* heterozygous pathogenic variants (Yates et al. 2017; Bramswig et al. 2017). These alterations can be identified through molecular genetic testing and exome sequencing (Leduc et al. 2017). The condition is characterised by moderate to severe developmental delay and intellectual disability, with speech and language delay as well as autistic features being common. Seizures have been reported in 95% of cases, often occurring under the age of two years (Balasubramanian 2022). Affected individuals are likely to be refractory to treatment, experience cardiac abnormalities, strabismus, renal anomalies, and skeletal features as well as abnormal breathing patterns, including hyperventilation and apnoea (Balasubramanian 2022; Unique 2019). The rarity of HNRNPU‐NDD and the variability in both presentation and severity of symptoms leads to diverse and unpredictable medical and social needs, making life with the condition particularly complex.

While several studies have sought to understand the genetic basis and phenotypic variance of the condition (Taylor et al. 2022; Rooney et al. 2023), there is to our knowledge no existing research into the experiences of families.

Existing research into other rare diseases that manifest in early childhood points to substantial and ongoing difficulties not only for the individual but also for their families (Boettcher et al. 2021; Baumbusch et al. 2019). For example, studies on neurodevelopmental and epileptic conditions have reported that seizure burden is a major source of parental stress (Berg et al. 2019). Studies suggest that parents, as primary caregivers to children with rare disorders, face significant emotional, physical, and financial difficulties in managing their child's condition (McMullan et al. 2021; Gómez‐Zúñiga et al. 2019). Furthermore, uncertainty surrounding disease progression can further contribute to psychological distress (Atkins and Padgett 2024; Dellve et al. 2006). Lack of information, guidance (Atkins and Padgett 2024; Dellve et al. 2006), and frequent delayed diagnoses (Zurynski et al. 2017) can lead to frustration and heightened stress for caregivers (Boettcher et al. 2021). Despite these challenges, some parents exhibit great resilience and positivity, drawing strength from their caregiving roles and supportive networks (Carpenter et al. 2018; Dellve et al. 2006).

Given the phenotypes of the condition, caring for a child with HNRNPU‐NDD could be particularly challenging for parents, who must navigate unpredictable health issues alongside day‐to‐day parenting responsibilities; but how parents experience this phenomenon has not been researched.

## 2 Methods

### 2.1 Design

Situated through phenomenology, hermeneutics, and idiography, Interpretive Phenomenological Analysis (IPA) is a qualitative method suited to exploring how individuals make sense of significant life experiences (Pietkiewicz and Smith 2014). Previous studies have successfully adopted IPA to investigate parenting experiences of children with other rare genetic conditions (e.g., Carpenter et al. 2018; Martin et al. 2023; Smith et al. 2006). As this is a novel research area and IPA is not hypothesis‐driven but instead seeks to explore how individuals perceive and make sense of their experiences it was chosen as the most suitable analysis method for this topic. Ethical approval was obtained from the University of Sheffield Medical School Ethics Committee. Participants were given the opportunity to de‐brief after the interviews with the second author, a registered Genetic counselor, if they felt affected by anything raised during the research process. The participants' experiences are presented to maintain confidentiality and privacy by removing any identifiers in the data such as the child's sex and age, number of children and geographical location.

### 2.2 Participants

Participants were recruited through purposive sampling, specifically targeting parents with children diagnosed with HNRNPU‐related disorder. To be eligible, participants needed to be fluent in English and a parent or guardian of a diagnosed child and capable of providing informed consent. The study aimed to conduct and analyze between 4 and 10 interviews, over a six‐month period in line with guidelines for IPA by Pietkiewicz and Smith (2014).

An open invitation recruitment poster containing the researchers' contact details was uploaded to the international BF4U HNRNPU Family Foundation Facebook support group by the group administration. Those who expressed interest were sent a detailed participant information sheet. Written consent was obtained from each participant prior to their inclusion in the study. Participants were informed of their right to withdraw at any time without consequences. Seven participants who met the study's criteria consented to be interviewed.

Despite open and inclusive strategies used to recruit participants, unfortunately no fathers responded to the research study by advertisement. All seven of the participants were biological mothers, shifting the focus of this research from ‘parents’ to specifically mothers. Six were in a committed relationship with the child's father; one was divorced and living alone. Participants lived in the UK, USA or Australia. The age of their child with HNRNPU‐NDD ranged from 13 months to 23 years. Among the seven children, three were female and four were male. All were the youngest child in their family; three were only children while four had older siblings. Pseudonyms were assigned to participants to preserve anonymity: Erin, Ellie, Molly, Sophie, Rebecca, Hannah and Charlotte. Four participants (Erin, Ellie, Molly and Sophie) all had more than one child, whereas three (Rebecca, Hannah and Charlotte) had only one child. To preserve anonymity in this ultra‐rare condition, identifying details such as the child's age and specific geographical location have not been included. While the authors appreciate that children's age could be an important contextual factor, maintaining participant anonymity was a key ethical priority in this study.

### 2.3 Data Collection

Semi‐structured interviews were conducted by the first author online via Google Meet, allowing for flexible and accessible participation across the different geographic regions. Each interview was guided by a set of 16 open‐ended questions, as outlined by Pietkiewicz and Smith (2014) and Eatough and Smith (2017) (Appendix A1). Additional follow‐up questions were asked to explore points raised by participants, who were encouraged to clarify their responses where necessary. The interviews ranged in length from 25 min to 1 h and were conducted between April and June 2024.

### 2.4 Analysis

The analysis was conducted following the most recent guidelines for IPA as outlined in Smith et al. (2021). The creation and naming of themes was guided by suggestions in Smith et al. (2021) and Nigbur and Chatfield (2025).

The process consisted of several stages. Step one: to maintain the accuracy and immediacy of the data, interviews were transcribed immediately by the first author after they took place. The transcript was read multiple times, allowing the researcher to engage deeply with the data. Step two: initial ideas and interpretations were noted in the margins. Step three: these notes and interpretations were developed into Experiential Statements, phrases grounded in the participants' own words that reflected individual insights and experiences. Step four: these Experiential Statements were organized into clusters where meaning overlapped and connected to form Personal Experiential Statements (PETs). Step five: a table of PETs was constructed for the participant and emergent topics and ideas related to the interview were bracketed, meaning they were set aside to avoid bias and leading questions in further analyses to preserve an idiographic focus (Smith et al. 2021).

Step six: steps one to five were repeated for each participant. Step seven: when all of the interviews had been analyzed and a table of PETs had been constructed for each participant, PETs across participants were grouped into Group experiential Themes (GETs). The GETs highlighted shared patterns across multiple participants where meaning converged but still sought to remain idiographic and respect individual nuance.

### 2.5 Rigor/Trustworthiness

The interviewer maintained reflexivity throughout the research process via journaling, debriefs and regular supervision from other team members, ensuring that interpretations were closely aligned with participants' accounts rather than personal biases.

## 3 Results

Through interpretative phenomenological analysis, three interconnected group experiential themes (GETs) were developed: 1. From nurturer to care manager: how hypervigilance shapes daily life and maternal identity, 2. How mothers make sense of futures that no longer look as expected: grief, fear of future and reconstruction of hope, 3. Social worlds that ‘no longer fit’: experiences of isolation and disconnection.

### 3.1 From Nurturer to Care Manager: How Hypervigilance Shapes Daily Life and Maternal Identity

This theme describes how HNRNPU‐NDD does not replace motherhood with caregiving but introduces an additional layer of clinical responsibility that participants felt had to be incorporated into their maternal identity. Past and on‐going experiences of severe symptoms mold not only daily activities but mothers' perceptions of themselves and their role. The participants described how experiences with severe seizures, multiple emergency hospital admissions, and the general unpredictable nature of the condition lead to a state of hypervigilance.

Hannah described watching her child “like a hawk” and for many participants this hypervigilance dictates their daily lives:

I used to drop her off [at kindergarten] and then I'd come home and I'd go for a walk, that was like my mental health thing. And now I'm realising that I've been too afraid to do that because I'm afraid of being a couple miles from home, having daycare call and not being able to get there in a hurry. I realised that my day‐to‐day was changing […] I'm afraid to be too far away in case something happens.

Here hypervigilance and lifestyle changes gradually crept up on Hannah. She no longer feels able to take walks in fear of being too far away if something happens to her daughter. The issue isn't just that she worries about her child, but her own freedom is limited because she's *“afraid”* of being out of physical reach in case an unpredictable medical event occurs. She states that she is *“now realizing”* how hyper‐aware she has become. Her child's potential medical needs restructure her own routine and access to wellbeing activities.

Erin similarly described how the demands of managing her child's condition had extended her maternal role beyond everyday caregiving into the coordination of complex and ongoing care:

So he doesn't go to daycare, I stay at home. My husband will give him medicine in the morning but usually I'm the one running him to therapies. I mean everything, the medicines. He's on a keto diet for his epilepsy. I do all the keto cooking…I think I will probably die early from a heart attack or something, haha… I think it's just really hard on your health. (Erin)

Erin structures her narrative around the build up of responsibilities. Although participants had mixed levels of support from their co‐parents, Erin distinguishes her own role as encompassing *“everything”*, including coordinating therapies, managing medication and preparing specialist diets for her child's epilepsy. Her repeated use of *“I”* positions her as the central organiser of this care. These responsibilities go beyond anticipating the everyday needs commonly associated with motherhood, requiring her to incorporate ongoing medical and therapeutic management into daily family life. Her remark that she will *“probably die early from a heart attack or something”* followed by laughter, acknowledges the perceived toll of sustaining these responsibilities. Rather than presenting self‐sacrifice as an inherent feature of motherhood, she suggests the intensity and complex nature of her child's care had extended what she felt responsible for, with consequences for her own wellbeing.

Severe symptoms, their unpredictability and the ongoing demands of managing HNRNPU‐NDD meant that for some participants, the experience of motherhood involved more than the day‐to‐day care of the child. For Molly, these demands were experienced as a distinct shift between being a *“mother”* and a *“caregiver”*:

My brain switched from mother to caregiver. So I couldn't get emotionally attached. I had to separate myself from who she was, to know what I had to do for her. That was hard and that was difficult. (Molly)

Molly explicitly distinguishes between the identities: *“mother”* and *“caregiver”* describing her brain as having *“switched”* from one to the other. The caregiver role appears to require a different orientation towards her daughter: Rather than being emotionally attached, she felt she had to *“separate”* herself and focus on *“what [she] had to do for them [child].”* The distinction she draws suggests that the practical and medical demands of care could, at times, take priority over the emotionally nurturing aspects she associated with being a mother. Unlike Hannah, whose shift towards hypervigilance seemed gradual, Molly describes a more conscious shift in how she positions herself in response to her child's needs. Describing it as *“hard”* and *“difficult”* indicates the tension involved in sustaining both roles. The demands of caregiving required her to temporarily alter how she enacted her maternal role.

This expansion of the maternal role was also reflected in the need to develop knowledge and advocate for their child within healthcare settings. As Rebecca explained:

you do have to become the expert and become your child's advocate. (Rebecca)

The use of *“become”* is significant, suggesting that *“expert”* and *“advocate”* were not roles she automatically possessed, but identities that developed in response to her child's condition. Here motherhood involves a process of learning and adaptation, through which intimate knowledge of her child is accompanied by responsibility for managing services and advocating for specific needs. In this sense, participants were expected to acquire and use knowledge in ways to question, negotiate and advocate within the context of their child's healthcare.

The need to be proactive was also described as a way of negotiating the uncertainty surrounding the condition

We want to do what we can… I'm proactive… I like to try to act like I have some control (Hannah)

Hannah's phrasing, *“I try to act like I have some control,”* acknowledges the limits of what she can control. In the context of an unpredictable condition, being proactive offers a way of responding to uncertainty. It becomes a way of managing uncertainty and sense of agency in circumstances where participants could not predict or prevent what might happen to their child.

However, sustaining these expanding responsibilities came at a personal cost. Rebecca described the impact on her own wellbeing:

I'm in lots of therapy. I am medicated. I have good days and bad days. The example that I like to use is that some days I wear a cape and I'm super mom and some days I have to hide under my cape and I can't get out for a few days.

Rebecca's account highlights the difficulty of sustaining the expectations associated with being a mother, the demands of caring for her child while also managing her own wellbeing. The contrast between being *“super mom”* and needing to *“hide under [her] cape”* illustrates the tension between continuing to carry out caregiving responsibilities and recognising her own limits. The *“cape”* can therefore be seen to represent the expectation to remain capable, proactive and responsive, while hiding beneath it reflects times when the emotional toll of sustaining this role became overwhelming.

### 3.2 How Mothers Make Sense of Futures That No Longer Look as Expected: Grief, Fear of Future and Reconstruction of Hope

Participants described a shift in how they imagined their child's future following an HNRNPU‐NDD diagnosis. Across interviews participants reflected on the loss of previously held expectations about their child's development, independence and adulthood, meaning they have had to reconsider “*hopes and dreams”* about what the future might look like. Whilst some mothers described this process in terms of grief and mourning, others spoke more about uncertainty, fear, or hope. These emotional states were often experienced alongside one another. Mothers moved between distress about what had been lost, anxiety about what remained unknown, and attempts to imagine new possibilities for the future.

Sophie explicitly acknowledges her experience as one of *“grieving”*:

There's an element of grieving when you have a child like this […] and you begin to accept this is lifelong. This is forever for this child […] It's quite hard to come to terms with because you have so many hopes and expectations.

She seems to mourn the loss of *“hopes and expectations”* associated with rare‐disease parenthood. The emphasis on *“lifelong”* and *“forever”* highlights a permanent impact, her grief intensifies as the reality of the condition becomes more difficult to imagine.

Sophie's interview demonstrated a process of grieving altered expectations. Reflecting on her son's development and seeking medical support, she explained:

We pressed on regardless. I knew he was never going to catch up. I knew.”/“It was a drip drip of just coming to terms

Although Sophie initially *“pressed on regardless,”* showing perseverance and resilience, the repetition of *“I knew”* creates a sense of certainty and inevitability. It suggests she had already begun mourning the future she had originally imagined for her son. Her interview showed a gradual realisation that expected milestones (*“he was never going to catch up”*) may never be reached. This demonstrates future‐orientated grief, grieving the loss of an expected developmental future. Sophie described the diagnosis as *“a drip drip of just coming to terms.”* The metaphor of a *“drip drip”* suggests something slow and repetitive. Grief is something that gradually wears her down over time. For Sophie this adjustment is connected to the loss of previously imagined futures.

Hannah described a similar process of future‐orientated grief but also brings in specific anticipatory grief for the loss of her child's life:

I'm very worried about the seizures and just want to feel safe in what our current treatment plan is, and we're just not there yet…I have a hard time not knowing. I feel despair at times because I worry about her future and this obviously isn't anything we ever expected and in a way there's anticipatory grief for potentially having to mourn the loss of life, dreams and visions that we had for her but we'll come up with new dreams and visions and I know that she's going to be fine.

Hannah also acknowledges grieving the loss of an imagined future, however, her choice of language differs. She uses anticipatory grief to describe the specific fear she has about poor seizure management causing the loss of her child's life. However despite this, while Hannah describes feelings of *“despair”*, she simultaneously has a more active engagement with future possibilities compared to Sophie who focused on what had been lost. Hannah holds grief and hope alongside one another. The phrase *“dreams and visions”* is repeated twice; initially she is referring to what may be lost, but later this becomes something that can be recreated or reimagined.

Hannah also revealed the emotional difficulty of living with uncertainty. She also described feeling *“terrified all the time”* and “*worry[ing] about the future.”* Similar to Sophie, concerns about the future go beyond practical considerations and become emotionally consuming. For Sophie, however, uncertainty developed into a deeper sense of hopelessness. She described her future as *“a miserable prospect”* and questioned, “*Why am I bothering to get up in the morning if this is what our future is?”* The rhetorical nature shows emotional exhaustion and defeat; it suggests that uncertainty about her son's future is affecting her own sense of purpose. Her use of *“our future”* further highlights how closely her own identity and imagined future has become tied to that of her child's (GET 1).

Concerns about the future were evident throughout Ellie's interview, although her narrative was expressed through fears about her daughter's vulnerability. She described feeling *“in depression constantly” and “I just want out of misery, for her and for myself,”* suggesting that her own emotional wellbeing is intertwined with concerns about her daughter's future. This became more apparent when she reflected:

My biggest fear is that someone will abuse her and she won't even know […] that is what keeps me up at night. A future where my husband and I are no longer alive and she's alone.

Unlike Sophie whose distress focused on a future that felt hopeless, Ellie imagined future scenarios that she found frightening. Her fears focused on possibilities of her daughter being left without protection. The phrase *“keeps me up at night”* demonstrates how emotionally intrusive these concerns had become. Alongside these fears, Ellie also articulated hope:

Somehow we'll figure it out. She'll go to college and she'll be independent. And so I'm unwilling to take no for an answer, in terms of her progress.

Like Hannah, Ellie moved between fear and hope, suggesting that these emotions coexist. Whilst Hannah's hope involved imagining new *“dreams and visions,”* Ellie's hope feels tied to determination and effort because she is *“unwilling to take no for an answer.”* Her perspective illuminates both anxiety about the future and a drive to secure a more positive future for her daughter.

While Hannah and Ellie move between fear and hope, Charlotte demonstrated that these are part of the same experience, each feeding into the other:

There are times where I feel really optimistic… and other times I'm half optimistic and half worried. (Charlotte)

### 3.3 Social Worlds That ‘no longer fit’: Experiences of Isolation and Disconnection

Social landscapes were reshaped by their child's diagnosis and the ongoing care demands. Friendships, communities, and shared experiences that were once familiar and grounding now feel limited. For several mothers, everyday interactions with other parents became sources of discomfort and emotional strain rather than connection.

Erin found herself disconnecting from social interactions with other parents because their concerns seemed trivial to her own; she compared realities:

I don't really like socialising anymore because it's hard […] parents will be complaining ‘oh my kid has a fever, it's terrible’ and, it's hard to be compassionate with what I'm going through. (Erin)

The contrast Erin draws between a common temporary illness and her own experience of her son's condition suggests parenting conversations are no longer relatable. Her difficulty being *“compassionate”* doesn't necessarily indicate a lack of empathy, but it highlights the emotional distance she puts between her own reality and other parents. Social interactions become reminders of how different her current circumstances feel.

Ellie also finds it hard to have shared understandings of parenthood. She previously related to other parents through her older neurotypical child, but her experiences have shifted since having a child with HNRNPU‐NDD:

Its very difficult for me to have conversations with parents who typically don't identify or are not on the same wavelength as me.

Ellie's narrative however is characterized by choice rather than emotionally distancing herself. She stated:

I don't have friends anymore. That's intentional because all of my friends have neurotypical kids and then I just get very upset when I interact.

She uses *“that's intentional”*, which marks her withdrawal as deliberate. She actively distances herself from certain relationships. Her interactions with other parents are emotionally difficult and she uses avoidance as a form of emotional protection.

Molly, on the other hand, felt that social disconnection was more of a gradual consequence of the caregiving demands:

I have lost friends. We don't get invitations anymore. I think it's because through the years we've had to cancel so many times. (Molly)

Unlike Ellie who intentionally withdrew, Molly's relationships became increasingly difficult to maintain due to the unpredictability of her child's condition. *“We don't get invitations anymore”* suggests a sense of loss and exclusion. Several other mothers described feeling detached from the everyday social worlds they once had because they no longer seem to accommodate their reality. Sophie described it as *“isolating to have a child with a condition nobody understands”* and reflected on how this sense of isolation surfaced not only among peers but also in her interactions with professionals:

The hospital couldn't tell us anything… Nobody knew anything about it. Nobody knew what to do about it. Nobody could offer any real help… The social workers, they've never heard of it so they don't know what you're talking about… (Sophie)

Despite her lived expertise, it holds little authority, leaving feelings of frustration and invisibility in systems meant to support them.

The ability that I have encountered for people to ignore my input… It's frustrating to have the condition constantly disregarded.

The emotional weight of being unheard underlines the loneliness already felt through the loss of social connection. This resulted in feelings of sadness and loss of hope (GET 2).

In contrast, Rebecca and Hannah described how online and rare disease communities provided opportunities to connect with families who share an understanding of HNRNPU‐NDD:

The HNRNPU Facebook group and families have been fantastic […] They have been a huge help […] We went to Disney World with one of the families (Rebecca)

Rebecca suggests that these connections create relationships and shared social experiences that are difficult to find. Going to Disney World with another family illustrates how connection through the HNRNPU community can be more than just exchanging information about the condition and can provide a sense of belonging based on shared experience.

For Hannah, the value of these communities is being able to speak with people who understand aspects of the condition that others cannot:

The Facebook groups have been really helpful to have people who understand the diagnosis and the rarity of it and all the things that come with that to talk to and answer questions. (Hannah)

Support offered by these communities are grounded in shared experiential knowledge. These spaces bring together people who already understand the condition and Hannah doesn't need to explain or justify her child's needs. The groups therefore appear to offer not only practical opportunities to *“answer questions”* but as a form of recognition, in which participants' experiences can be understood.

Within these networks, access to other families' experiences also exposed participants to overwhelming aspects of the condition:

I joined these Facebook groups… the real‐life experiences… have been huge… but it can be overwhelming (Hannah)

The same shared experiences that made these groups valuable also confronted Hannah with the realities of other families' lives with HNRNPU‐NDD. Therefore, connection doesn't always alleviate isolation, it also increases awareness of the potential severity and difficulties of the condition. For Hannah, accessing a community of people who understood her experience meant gaining both support and increased exposure to the uncertainties she was trying to navigate.

## 4 Discussion

The results of this research provide insight into how the unique uncertainties and rarity of HNRNPU‐NDD shape maternal sense of self, emotional life and patterns of social connection. While existing literature has examined experiences of parents caring for children with rare disorders and similar conditions (Anderson et al. 2013; Baumbusch et al. 2019; Cooper et al. 2024; Davidson et al. 2023), this study to our knowledge is the first to focus specifically on HNRNPU‐NDD.

In the first theme, gendered expectations were evident despite varied levels of partner involvement. Mothers assumed the primary responsibility for coordinating care, which reflects longstanding findings that mothers tend to take on the majority of caregiving tasks in contexts of disability (Carbone et al. 2024; Gray 2003). Dellve et al.'s (2006) prospective study followed parents over 12 months after a family‐competence intervention and found that mothers reported significantly higher psychological and physical strain than fathers. While Dellve et al. captured these patterns through standardised measures, the present IPA analysis adds experiential depth by illustrating the profound impact that caregiving demands had on maternal wellbeing. Mothers' accounts of hypervigilance, emotional strain, and restricted freedom provide rich insight into the lived experience of caring for a child with HNRNPU‐NDD. However, because fathers were not included in this study, future research is needed to explore whether similar impacts are experienced across different caregiving roles.

For some mothers, the demands of caring for a child with HNRNPU‐NDD created a distinction between being a *“mother”* and a *“caregiver.”* Davidson et al. (2023) similarly found that parents of children with rare diseases had to navigate the demands of being both a parent and a caregiver. However, in the present study, this distinction was shaped by the severity and unpredictability of HNRNPU‐NDD. The possibility of severe seizures and other unpredictable symptoms meant that caregiving heavily relied on constant vigilance and being prepared to respond if something happened. This may make it more difficult to separate the roles of mother and caregiver, as the demands of the condition could dictate many aspects of everyday life.

While hypervigilance, anticipation of a child's needs, and self‐sacrifice may be culturally associated with normative motherhood, participants described these expectations as becoming intensified by the severity and unpredictability of HNRNPU‐NDD. Mothers described remaining in a constant state of readiness, with clinical monitoring becoming embedded within everyday parenting practices. Advocacy and role restriction have also been reported for parents with children across a range of childhood disabilities and rare conditions, as well as the toll this can take on maternal mental health (Dellve et al. 2006; Docherty and Dimond 2017; Gray 2003; Pelentsov et al. 2016; von der Lippe et al. 2022). The present findings show that these responsibilities not only added to but altered how participants understood motherhood, creating tension between being emotionally present as a mother and functioning as the vigilant caregiver, coordinator, expert, and advocate that their child's condition required. This highlights the need for clinical services to recognise not only caregiving burden but also the sustained psychological demands associated with managing ongoing medical uncertainty in HNRNPU‐NDD.

In the second theme participants described the condition as fundamentally altering how they imagined and understood the future. Uncertainty surrounding managing symptoms, development and long‐term independence frequently emerges as a challenge for parents of children with rare genetic disorders (von der Lippe et al. 2022; Weng et al. 2011; Somanadhan and Larkin 2016).

Although uncertainty was a shared theme, mothers experienced it in different ways. Uncertainty appeared to generate a range of responses including grief, anticipatory loss, fear, and hope. For some participants, hope and fear appeared alongside one another, aligning with Cooper et al.'s (2024) observation that individuals at risk of Huntington's disease face a constant movement between emotional worlds without fully resolving the tension between them. These findings suggest that adapting to a HNRNPU‐NDD diagnosis in a child is an on‐going process of negotiating uncertainty and not a linear process of adjustment and acceptance.

Hemati Alamdarloo and Majidi (2020) found elevated levels of future‐focused hopelessness among mothers caring for children with neurodevelopmental disorders. Their work suggests that uncertainty about developmental progress and long‐term independence can affect how parents think about the future. In this study, hopelessness was not described as a fixed feeling; instead, it came and went, often resurfacing during periods of medical instability or when mothers thought about their child's adulthood or care after they themselves are no longer able to provide it. This shifting emotional landscape reflects how deeply mothers are trying to make sense of an uncertain future while still finding motivation and meaning in the present (Berrios et al. 2024; Turbitt et al. 2024). Hope, on the other hand, seemed to emerge through determination, persistence, and agency rather than certainty. It functioned as a way of responding to uncertainty. Mothers described advocating for their children; as discussed in GET 1, advocacy served a practical and emotional purpose as a way of coping with uncertainty and sustaining hope.

The findings also suggest links between experience of altered futures and wider social experiences. Interestingly, we found that mothers who described positive experiences with rare disease communities seemed better able to maintain hope alongside uncertainty compared to accounts characterised by greater social and professional engagement. This suggests that connection and belonging may play an important role in how mothers navigate the emotional challenges of imagining uncertain futures.

Finally, the third theme captured the social consequences of raising a child with HNRNPU‐NDD. Participants frequently expressed feelings of isolation. Although social isolation is commonly reported among parents of children with rare and complex diseases (Currie and Szabo 2020; Baumbusch et al.'s 2019), the ultra‐rarity of HNRNPU‐NDD may further contribute to this disconnection. With the condition only relatively recently recognised and a small number of diagnosed individuals worldwide, opportunities for mothers to meet others with direct knowledge of the condition may be limited (Balasubramanian 2022).

The purposeful withdrawal that some participants made from their existing social worlds was intentional as it created a sense of emotional safety and served as a means for protection from difficult interactions. While others mentioned being forced out due to caregiving demands and conflicting responsibilities. Regardless, the loss of social worlds appeared to represent more than a reduction in social contact, it signified a significant emotional loss beyond individual relationships to mothers' sense of belonging and connection to others. This may also be understood in the context of altered futures mothers described (GET 2), as coming to terms with a child's diagnosis involves reconsidering not only their expectations for their child's life but also their own as parents. Such changes may have further shaped mothers' experiences of social connection, particularly where existing relationships and social environments no longer aligned with their changing circumstances, and contributed to feelings of disconnection.

Disconnection also seemed to appear within healthcare interactions. Limited awareness of rare conditions among healthcare professionals has been widely reported (Pelentsov et al. 2015, Baynam et al. 2024 and Currie and Szabo 2020) but the present findings suggest that the consequences of this extend beyond practical challenges because mothers described feeling unheard and disregarded despite having considerable knowledge. This contributes to a deeper sense of isolation and disconnection and highlights the importance of recognising parent's experiential knowledge.

Online rare‐disease communities provided mothers with opportunities to connect with people. These spaces appeared to enhance practical guidance or information given to them by professionals as well as providing a sense of belonging in which mothers could share fears and uncertainties without needing to repeatedly explain their child's condition. Condition‐specific peer groups have previously been recognized as an important source of emotional and informational support (Lasker et al. 2005; McMullan et al. 2020) and are particularly valuable in the context of an ultra‐rare condition such as HNRNPU‐NDD, where opportunities for shared understanding may be difficult to find within existing social networks.

Furthermore, Bogart and Hemmesch (2016) found that attending in‐person conferences and support groups provided parents with opportunities to connect with others who share similar experiences and gain knowledge. However, for ultra‐rare conditions like HNRNPU‐NDD, these events are less common and often geographically or financially inaccessible to many families. Even when mothers are able to access such spaces, they may be confronted with the diverse and sometimes more severe trajectories of other children.

Indeed the findings of this study also suggest that peer support is complex and while these communities can reduce isolation, they also expose mothers to the variability and difficult realities of living with HNRNPU‐NDD. Shared experience therefore can provide reassurance and validation while simultaneously intensifying the uncertainty surrounding the condition. Within this landscape, genetic counselors can help to mitigate these effects through clearer communication between families and healthcare providers as well as their role in offering psychosocial support and directing families towards mental health and peer support resources.

## 5 Study Limitations

Firstly, it is important to note that participants are more likely to share negative experiences. This tendency likely results from varying levels of access to support and resources. Those already receiving adequate support may not feel the need to participate, while those lacking support might seek validation to be heard and acknowledged by highlighting negative experiences (Gouveia et al. 2022). This selection bias should be considered when interpreting the results.

This research focused exclusively on mothers; therefore, the perspectives of fathers or other male caregivers were not represented in this study. Whether fathers experience the same phenomena highlighted by the GETs identified in this study is an interesting avenue of research. This could be achieved by ensuring future research includes a more inclusive mix of both mothers and fathers or focuses specifically on fathers to ensure their perspective is understood. Furthermore, this study did not examine the broader family context. Previous studies have indicated that siblings of children with rare diseases are at higher risk of psychological challenges, particularly feelings of isolation and uncertainty (Haukeland et al. 2021). Future research could consider including siblings and other family members of those diagnosed with HNRNPU‐NDD to provide a better understanding of the impact of HNRNPU on the whole family unit.

In addition, all participants were residents of either the U.K, the United States of America, or Australia, countries with established genetic testing and genetic counseling services. Future research should seek to understand how the experiences of mothers/parents differ in different geographical and cultural contexts.

## 6 Research Recommendations

While the study itself is not considered a longitudinal study, which can be seen as a limitation, many of the experiences shared by participants span over extended periods ranging from over twenty years for some to just recently received a diagnosis for others. Molly remarked *“what I dealt with then and what I am dealing with now is very different”* which shows the importance of considering longitudinal data to fully capture changing circumstances for parents. Future studies could follow families over time to understand how their experiences and needs change as children with HNRNPU‐NDD grow older. This would provide insights into the long‐term impacts and types of support needed.

While the current findings offer valuable insights into the experiences of mothers and demonstrate a need for more support for this group, specific support packages or interventions to help mothers and families would require significant future research with a larger sample size incorporating mothers, fathers, and other family members from diverse contexts.

## 7 Implications for Practice

The complexity of these experiences, highlighted by the three GETs, is important to consider in genetic counseling. Allowing a space for mothers to explore the conflicting dichotomy of their experiences and how this shapes maternal identity needs careful facilitation. As genetic counselors, we are uniquely skilled to understand the complex medical nature of conditions like HNRNPU‐NDD whilst attending to the psychosocial impact of having a child with a rare genetic disorder. These themes encourage counselors to reflect on their own assumptions and biases, promoting reflexivity in practice. For example, assuming a parent is “coping well” because they appear organised may overlook the emotional cost of hypervigilance.

As rapid and expanding access to technologies like whole genome sequencing increases the diagnostic rate for rare conditions like HNRNPU‐NDD, the demand for genetic counseling expertise will expand. As we pick up from diagnoses made in mainstream settings to provide holistic care for families, a greater understanding of the lived experience of these rare and ultra‐rare conditions is vital.

## 8 Conclusion

This study delved into the experiences of seven mothers raising children with HNRNPU‐related neurodevelopmental disorder, offering one of the first in‐depth accounts of life with this ultra‐rare condition. Mothers described the constant work of navigating uncertainty, hypervigilance, and the emotional duality of holding hope alongside grief. They also spoke of significant changes in their social worlds, as familiar relationships no longer fit the realities of caring for a child with HNRNPU‐NDD. The findings highlight that the greatest challenges extend beyond symptom management. Mothers' accounts revealed that much of the difficulty lies in how they make sense of their roles, relationships and future, highlighting the need for support that recognises the emotional and relational realities of caregiving alongside the medical demands. Meaning‐making, advocacy and connection with others emerged as ways that mothers sustained hope and resilience.

At the same time, systemic factors shaped the extent of parents' burden. Limited clinical knowledge, fragmented care pathways and the absence of psychological support for some intensified the pressures mothers described. Genetic counselors are well‐positioned to bridge these gaps through clear communication, advocacy within healthcare settings and guidance towards mental health and peer support resources. Future research should include fathers, siblings and wider family members as well as families from diverse cultural and healthcare contexts to broaden understanding of how experiences differ across systems and identities. As awareness of HNRNPU‐NDD grows, it is important that services adapt to ensure they provide support that fully recognizes the complexity of their day‐to‐day life.

## Author Contributions

**I. Overton:** conceptialisation, investigation, formal analysis, data curation, writing. **F. Docherty:** supervision, methodology, writing. **M. Balasubramanian:** supervision, project administration, reviewing.

## Disclosure

Artificial intelligence (AI) tools were not used in the design, data collection, analysis, or writing of this manuscript.

## Ethics Statement

Human studies consent: ethics approval for this study was obtained via the University of Sheffield Medical School Ethics Committee. Informed consent was obtained from all participants prior to their inclusion in the study.

## Conflicts of Interest

The authors declare no conflicts of interest.

## Data Availability

Authors elect not to share data.

## Interview Questions

1. How are you related to someone with HNRNPU?
2. Can you tell me a bit about you and your family?
3. How would you describe yourself/family?
4. Can you tell me about your child's symptoms?
5. How does your experience of caring for a child with these symptoms make you feel?
6. Has the severity of your child's symptoms changed with time? Have they gotten better/worse?
7. On a day‐to‐day basis, how do you manage these symptoms?
8. Can you tell me about your experience with existing treatment methods?
9. What are your views on the treatments?
10. Can you tell me about your experience of living with a child with HNRNPU?
11. Is there anything that stands out as particularly stressful?
12. What are some other effects of your experience of this diagnosis on your life?
13. Do you feel this diagnosis has an influence on your daily life?
14. What have you found useful in terms of providing support to you and your family? What have you found is helpful?
15. How do you feel these experiences may affect your life in the future?
16. What advice would you give to other families who have children with HNRNPU?
